# Maintaining Energy: A Potential Transformative Power to Adapt to the Challenges of Older Age?

**DOI:** 10.1093/geroni/igab046.1430

**Published:** 2021-12-17

**Authors:** Rebecca Ehrenkranz

## Abstract

Reduced energy is a hallmark feature of aging. Maintaining higher energy late in life may be a key adaptive strategy to the challenges that accompany older age and ultimately promote resilience. Perceived lack of energy is often construed as synonymous with fatigue, and energy and fatigue are frequently considered opposite aspects of the same phenomenon. However, evidence suggests that energy and fatigue have distinct underlying neurobiology. Further exploration of the energy/fatigue dichotomy is needed in community-dwelling older adults free of neuropathologies and clinically overt conditions. This symposium will first present clinical and epidemiologic justifications for operationalizing energy as a separate construct from fatigue and then will provide evidence on the underlying neurobiological correlates. Taken together, our results suggest perceived energy: a) overlaps with but is distinct from lower fatigability (Katz); b) may signal resilience against age-related declining mood and gait speed despite self-reported tiredness (Ehrenkranz); c) appear negatively influenced by Alzheimer’s neuropathology (Dougherty); and d) may reflect a distinct spatial distribution of brain functional connectivity (Hengenius). Thus, this symposium will explore energy as a mechanism related to yet distinct from fatigue and its implications for both healthy aging and neuropathological processes.

